# Impact of Sleep Health Domains on Chronic Conditions: Findings from a Cross-Sectional Analysis Using Data from the NHANES 2017–2020

**DOI:** 10.1055/s-0046-1824545

**Published:** 2026-07-17

**Authors:** Allan Saj Porcacchia, Julia Ribeiro da Silva Vallim, Monica L. Andersen, Vânia D'Almeida, Sergio Tufik

**Affiliations:** 1 Departamento de Psicobiologia, Universidade Federal de São Paulo, São Paulo, SP, Brazil; 2 Instituto do Sono, Associação Fundo Incentivo à Pesquisa (AFIP), São Paulo, SP, Brazil

**Keywords:** sleep, sleep apnea syndrome, sleep initiation and maintenance disorders, sleepiness, comorbidities, chronotype

## Abstract

**Objective:**

To examine the effect of aspects of sleep health on the likelihood of the occurrence of major public health outcomes.

**Materials and Methods:**

This is a cross-sectional study which analyzed data from 2 editions of the National Health and Nutrition Examination Survey (NHANES; 2017–March 2020), a nationally-representative survey from the United States, with a final sample size ranging from 7,361 to 7,373 participants. Five domains of sleep health were evaluated: symptoms of sleep breathing disorders, symptoms of other sleep disorders, sleepiness, circadian misalignment (social jetlag), and chronotype (mid-sleep on free days corrected for sleep debt on workdays). The primary outcomes were the odds of being diagnosed with cardiovascular and respiratory diseases, thyroid disorders, stroke, and cancer. Binary logistic regression models were used for data analysis.

**Results:**

The mean age of the participants was of 49.94 years. Regarding the sleep-related variables, 27.5% of the respondents reported difficulties with sleep, 47.4%, snoring, 12.3%, episodes of breathing cessation during sleep, and 25%, symptoms of daytime sleepiness. The mean social jetlag was of approximately 3 hours. Difficulties with sleep were connected with a higher odds ratio of all outcomes. Greater monthly frequency of daytime sleepiness was significantly associated with increased probability of presenting all chronic conditions. Later chronotype was related to an increased probability of developing thyroid, cardiovascular and respiratory diseases. Cessation of breathing during sleep was linked to cardiovascular and respiratory health issues.

**Conclusion:**

Impaired sleep health, particularly insomnia and sleep apnea symptoms, as well as later chronotype, were significantly associated with an increased likelihood of being diagnosed with all the outcomes tested.

## Introduction

The concept of sleep health is not solely defined by the absence of sleep disorders; rather, it is a multidimensional construct characterized by measurable sleep-related variables that significantly influence quality of life. The dimensions that are usually considered to comprise sleep health are sleep duration, efficiency, timing, quality, and regularity (including circadian aspects). 1 2 Given the critical role of sleep in overall well-being, there is a growing global movement to promote it as a fundamental pillar of health. 3

Sleep impairment is responsible for a significant economic burden and adversely affects the well-being of millions of individuals. It is the consequence of a combination of sleep disorders, insufficient, irregular sleep, and poor sleep quality, due to a range of lifestyle, socioeconomic, cultural, demographic, environmental, and other factors. This has led to sleep issues being classified as a neglected public health issue. 4

According to data from the 2020 National Health Interview Survey, 5 almost 2 out of every 10 adults in the United States (US) report difficulties initiating or maintaining sleep, and 12% are diagnosed with chronic insomnia. 6 Globally, approximately 936 million individuals suffer from obstructive sleep apnea (OSA), with China, the US, Brazil, and India recording the highest prevalences. 7 In Brazil, more than 30% of individuals are affected by OSA, with older age, male gender, and obesity conferring a greater risk of developing this condition. 8 9

In addition to sleep disorders and associated complaints, circadian health issues, such as social jetlag (SJL), are also highly prevalent. Defined as the discrepancy in sleep timing between work and free days, SJL is an important indicator of misalignment between an individual's timing system and their social schedule. 10 11 In the largest city in Latin America, São Paulo, Brazil, approximately 7 out of 10 individuals have experienced some level of it, with an estimated prevalence of high SJL (≥ 2 hours) of 10 to 14%. 12 13 Irregular sleep patterns can have a detrimental impact on health, 14 and there is evidence that SJL is more pronounced in individuals with later chronotypes. 15

Sleep disturbances, particularly OSA, insomnia, and SJL, have been linked to adverse health outcomes, including cardiovascular 16 17 18 and metabolic diseases, 19 20 stroke, 16 21 22 hormonal imbalances, 23 24 and cancer. 25 26 Despite the growing acknowledgement of the importance of sleep, there is a scarcity of studies that evaluate the relationship between sleep health domains and these outcomes in a comprehensive manner using large, representative samples. This deficiency in the existing literature highlights the need for broader investigations into the impact of sleep dimensions on public health.

Given this scenario, and the availability of a substantial sample from the US National Health and Nutrition Examination Survey (NHANES), the current study planned to conduct a cross-sectional analysis. The aim was to assess the associations involving various sleep health domains, specifically, self-reported symptoms of sleep disorder, circadian misalignment (SJL), and chronotype (mid-sleep on free days corrected for sleep debt on workdays, MSF _sc_ ). These sleep variables were examined in relation to five major public health outcomes: cardiovascular and respiratory diseases, stroke, thyroid problems, and cancer.

## Materials and Methods

### Study Population – NHANES

The data were extracted from the US NHANES, a core initiative of the Centers for Disease Control and Prevention (CDC), managed by the National Center for Health Statistics. The survey's objective is to evaluate the health and nutritional status of the civilian, non-institutionalized population. Through comprehensive methods, such as structured interviews, detailed physical examinations, and assessments of physical fitness, the NHANES gathers data annually from a nationally-representative sample of about five thousand individuals. It spans various age groups and geographic regions, thus providing critical health information. 27 Data from this study is made publicly available in sets every two years. All participants sign consent forms regarding the use of their data. For the current study, we used combined data from the period spanning from 2017 to March 2020 (available from: https://wwwn.cdc.gov/nchs/nhanes/continuousnhanes/default.aspx?Cycle=2017-2020 ; accessed on November 11, 2024). The following data were extracted: demographic variables, anthropometric measures, as well as data about medical conditions, sleep, and smoking habits.

### Participants and Procedure for Data Selection

**Fig. 1:**
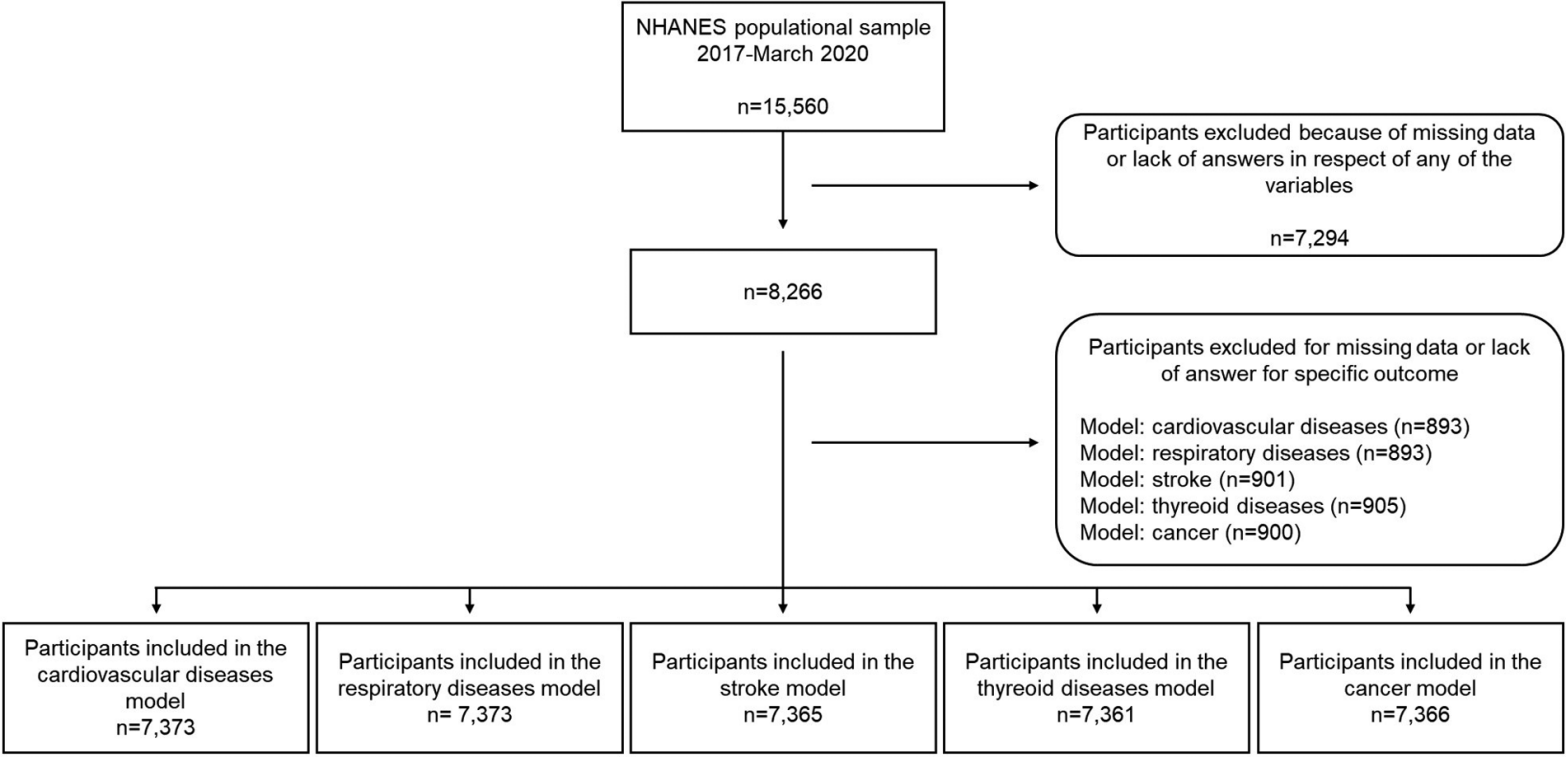
Flowchart of the data selection and the final sample size included in each binary logistic regression model.

The 2017–2020 NHANES database comprised 15,560 participants. From the total number of participants, those with missing data or who were under the age of 16 years were excluded from the analysis. The final sample size varied slightly, depending on the outcome evaluated in each model ( Fig. 1 ).

### Exposures, Outcomes and Statistical Analysis

A series of binary logistic regression models were developed to assess the potential relationships involving sleep parameters and the diagnosis of 5 distinct groups of chronic diseases: 1) cardiovascular diseases (congestive heart failure, coronary heart disease, angina, angina pectoris, or heart attack); 2) respiratory diseases (asthma, emphysema, chronic obstructive pulmonary disease, or complete heart block); 3) thyroid diseases; 4) stroke; and 5) cancer. The dependent variable for each analysis was the presence of a self-reported diagnosis of each of the aforementioned diseases at some point in the life of the participants.

The following sleep-related variables were included in the analysis: 1) frequency of snoring during the week (0–2 nights a week, ≥ 3 nights a week); 2) frequency of snorting or stopping breathing during the week (0–2 nights a week, ≥ 3 nights a week); 3) ever having told a doctor that they had trouble sleeping (yes/no); 4) frequency of excessive daytime sleepiness (never to 4 times a month, 5–15 times a month, 16–30 times a month); 5) circadian misalignment measured through SJL (continuous); and 6) chronotype assessed through the MSF _sc_ (continuous). Social jetlag was calculated as the absolute difference between mid-sleep on free days (Friday–Saturday and Saturday–Sunday) and mid-sleep on workdays (Sunday–Monday until Thursday–Friday). 10 The MSF _sc_ was calculated as described in the studies by Roenneberg et al., 15 28 correcting for longer sleep durations on free days compared to workdays. The chronotype classification was based on the same studies, 15 28 considering MSF _sc_ values equal or earlier than 3:00 AM as *early* , between 3:00 AM and 4:00 AM as *intermediate* , and later than 4:00 AM as *late* . All models were adjusted for gender, age, and body mass index (BMI). The one evaluating cancer was also controlled for smoking (a lifetime consumption of at least 100 cigarettes). Secondary models were additionally specified for each outcome, incorporating the poverty income ratio as a covariate to account for socioeconomic disparities in the sample. No sample weights were employed. The significance level was set at 5% ( *p* < 0.05). The statistical analyses were performed using the Jamovi (free and opensource; version 2.3) software, 29 30 31 and graphs were developed using the GraphPad Prism (GraphPad Software, Inc.) software, version 10.1.0. Further details regarding the collinearity of the variables included in the models can be found in Supplementary Material Table S1 .

## Results

Of the 15,560 participants from the initial NHANES 2017–March 2020 sample, between 7,361 and 7,373 individuals were included in the present study ( Fig. 1 ). There was a slight difference in the composition of the samples among the models. However, the descriptive analyses show that the mean values and CIs of the continuous variables and the relative frequencies of the categorical variables included were consistent regarding each analysis ( Tables 1 , 2 ). This enables the presentation of the descriptive data in a unified manner.

**Table 1:** Participants' characteristics regarding the continuous variables evaluated in the National Health and Nutrition Examination Survey (NHANES) 2017–2020.

	Mean + standard deviation (SD)	95%CI
*Cardiovascular diseases (N = 7,373)*		
Age (years)	49.94 ± 17.38	49.54–50.34
BMI (kg/m ^2^ )	29.97 ± 7.49	29.80–30.14
SJL	2.57 ± 3.80	2.49–2.66
MSF _sc_	3:08 ± 1:51 AM	3:05–3:11 AM
*Respiratory diseases (N = 7,373)*		
Age (years)	49.94 ± 17.38	49.54–50.34
BMI (kg/m ^2^ )	29.97 ± 7.49	29.80–30.14
SJL	2.57 ± 3.80	2.49–2.66
MSF _sc_	3:08 ± 1:51 AM	3:05–3:11 AM
*Stroke (N* *=* *7,365)*		
Age (years)	49.94 ± 17.38	49.54–50.33
BMI (kg/m ^2^ )	29.97 ± 7.48	29.80–30.14
SJL	2.58 ± 3.80	2.49–2.66
MSF _sc_	3:08 ± 1:48 AM	3:05–3:11 AM
*Thyroid diseases (N = 7,361)*		
Age (years)	49.94 ± 17.38	49.54–50.33
BMI (kg/m ^2^ )	29.97 ± 7.48	29.80–30.14
SJL	2.57 ± 3.80	2.49–2.66
MSF _sc_	3:08 ± 1:50 AM	3:05–3:11 AM
*Cancer (N = 7,366)*		
Age (years)	49.94 ± 17.38	49.54–50.33
BMI (kg/m ^2^ )	29.97 ± 7.49	29.80–30.14
SJL	2.57 ± 3.80	2.49–2.66
MSF _sc_	3:08 ± 1:50 AM	3:05–3:11 AM

**Abbreviations:** BMI, body mass index; MSF _sc_ , mid-sleep on free days corrected for sleep debt on workdays; SJL, social jetlag.

**Table 2:** Participants' characteristics regarding the categorical variables evaluated in the National Health and Nutrition Examination Survey (NHANES) 2017–2020.

	CV disorders: n (%)	RES disorders: n (%)	Stroke: n (%)	Thyroid disorders: n (%)	Cancer: n (%)
*Gender*					
Male	3,595 (48.8)	3,595 (48.8)	3,591 (48.8)	3,591 (48.8)	3,591 (48.8)
Female	3,778 (51.2)	3,778 (51.2)	3,774 (51.2)	3,770 (51.2)	3,775 (51.2)
*Frequency of snoring*					
0–2 night(s)/week	3,876 (52.6)	3,876 (52.6)	3,871 (52.6)	3,871 (52.6)	3,874 (52.6)
≥ 3 nights/week	3,497 (47.4)	3,497 (47.4)	3,494 (47.4)	3,490 (47.4)	3,492 (47.4)
*Frequency of stopping breathing/snorting*					
0–2 night(s)/week	6,463 (87.7)	6,463 (87.7)	6,458 (87.7)	6,455 (87.7)	6,459 (87.7)
≥ 3 nights/week	910 (12.3)	910 (12.3)	907 (12.3)	906 (12.3)	907 (12.3)
*Told doctor they had trouble sleeping*					
No	5,341 (72.4)	5,341 (72.4)	5,338 (72.5)	5,337 (72.5)	5,339 (72.5)
Yes	2,032 (27.6)	2,032 (27.6)	2,027 (27.5)	2,024 (27.5)	2,027 (27.5)
*Excessive diurnal sleepiness*					
0–4 times/month	5,542 (75.2)	5,542 (75.2)	5,539 (75.2)	5,537 (75.2)	5,539 (75.2)
5–15 times/month	1,269 (17.2)	1,269 (17.2)	1,266 (17.2)	1,264 (17.2)	1,268 (17.2)
16–30 times/month	562 (7.6)	562 (7.6)	560 (7.6)	560 (7.6)	559 (7.6)
*Outcome*					
No	6,749 (91.5)	5,863 (79.5)	7,025 (95.4)	6,521 (88.6)	6,634 (90.1)
Yes	624 (8.5)	1,510 (20.5)	340 (4.6)	840 (11.4)	732 (9.9)
Ratio of family income regarding poverty*					
Below poverty threshold	1,148 (17.9)	1,148 (17.9)	1,146 (17.9)	1,147 (17.9)	1,148 (17.9)
Average or above the poverty threshold	5,256 (82.1)	5,256 (82.1)	5,253 (82.1)	5,250 (82.1)	5,252 (82.1)

**Abbreviations:** CV, cardiovascular; RES, respiratory.

**Note:** *Missing related to ratio of family income regarding poverty: CV disorders – 969; RES disorders – 969; stroke – 966; thyroid disorders −964; and cancer – 966.

**Fig. 2:**
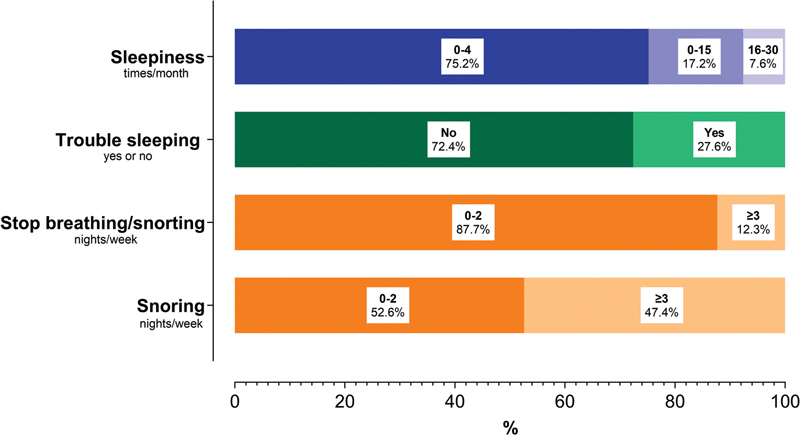
Proportion of self-reported answers for each sleep-related variable.

The mean age of the samples was of 49.94 ± 17.38 years, with 51.2% of the participants being female. The mean BMI was of 29.97 ± 7.49 kg/m ^2^ , indicating a tendency towards overweight, a factor related to several comorbidities. Fig. 2 presents information regarding the sleep domains in the samples. On average, the mean SJL was of 2.57 ± 3.80 hours, and the mean MSF _sc_ was at 3:10 ± 1:50 AM. The proportions of chronotypes in the samples were 55.5% of early, 23.1% of intermediate, and 21.4% of late. 15 28 In the independent evaluation of each regression model, the observed prevalence rates were as follows: respiratory disorders – 20.5%; thyroid diseases −11.4%; cancer −9.9%; cardiovascular disorders −8.5%; and stroke −4.6%. The results from the binary logistic regression models are presented independently for each model ( Supplementary Material Table S2 ).

### Cardiovascular Diseases

**Fig. 3:**
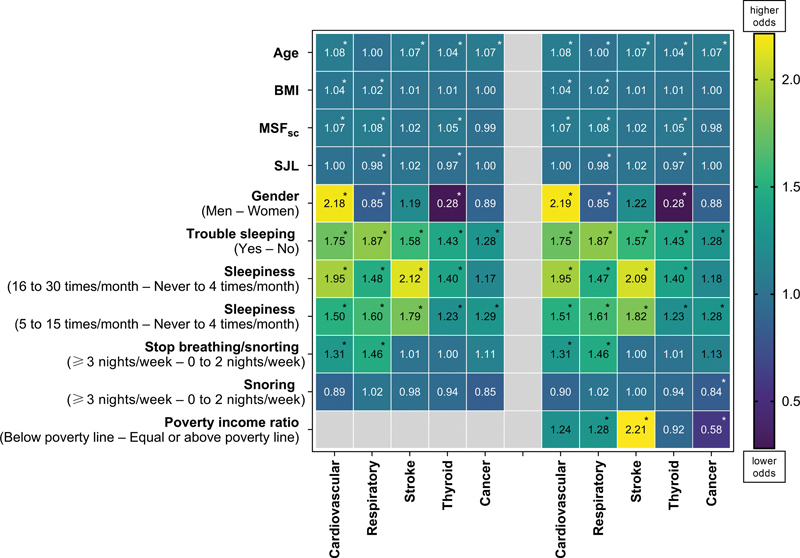
Heatmap of odds ratios resulting from the binary logistic regression models evaluating the association of demographic and sleep complaints with health outcomes in the National Health and Nutrition Examination Survey (NHANES) 2017–2020 sample. On the left, the models are not adjusted for the poverty income ratio; on the right, the models are estimated controlling for this socioeconomic indicator. The model evaluating cancer odds is also adjusted for smoking habits. Statistically significant results ( *p* < 0.05) are marked with an asterisk.

With the exception of SJL, snoring frequency, and poverty ratio, all other variables included in the model contributed to an increased odds ratio (OR) of being diagnosed with cardiovascular disease ( Fig. 3 ). On the unadjusted and adjusted for poverty income ratio models, the data indicated that male subjects had higher odds of having cardiovascular diseases. The sleep-related factors daytime sleepiness, trouble sleeping, and breathing cessation during sleep were all linked to an augmented likelihood of cardiovascular-disease diagnosis, as were older age, a higher BMI, and a later chronotype ( Fig. 3 ).

### Respiratory Diseases

In both regression models evaluating the factors associated with respiratory disorders, snoring frequency did reach significance ( Fig. 3 ). However, sleep complaints, including trouble sleeping, daytime sleepiness, and breathing cessation during sleep were associated factors that increased the chance of having respiratory disorders. The odds of having respiratory diseases were higher among female participants, those with a higher BMI, a later chronotype, and those with lower SJL. Being below the poverty line was tied to a higher probability of having respiratory diseases ( Fig. 3 ).

### Stroke

Neither gender nor BMI had a significant association with stroke. Advancing age contributed to a 1.07-fold increase in the odds of being diagnosed with stroke for each additional year ( Fig. 3 ). Among the sleep-related variables, excessive daytime sleepiness and trouble sleeping were significantly correlated with heightened stroke likelihood. Participants experiencing sleepiness and trouble sleeping had almost a two-fold increase in the likelihood of having a stroke. Those below the poverty line had higher odds of presenting the condition ( Fig. 3 ).

### Thyroid Diseases

Men were less likely to develop thyroid diseases ( Fig. 3 ). Older age contributed to an elevated OR, with each additional year increasing the odds by 1.04. Among sleep-related variables, the following, in descending order of probability, were found to increase the likelihood of developing thyroid disease: trouble sleeping, daytime sleepiness, and a later chronotype. Conversely, each additional hour of SJL corresponded to a reduction in it. The poverty income ratio, BMI, snoring or breathing cessation/snorting did not present statistically significant results ( Fig. 3 ).

### Cancer

Age, daytime sleepiness, and sleep complaints had significant associations with the increase in the likelihood of a cancer diagnosis ( Fig. 3 ). In the model adjusted for poverty income, higher snoring frequency was associated with lower odds. This result was not observed in the unadjusted model. Individuals below the poverty line were less likely to have this condition ( Fig. 3 ).

## Discussion

Through statistical models adjusted for the poverty income ratio, we identified that age was associated with all health outcomes: cardiovascular respiratory, and thyroid diseases, stroke, and cancer, which is consistent with the existing literature. 32 33 34 We observed a connection between BMI and cardiovascular and respiratory diseases. However, BMI was not related to thyroid diseases, cancer or stroke, although it is considered a risk factor for these conditions in the existing literature. 35 This may be due to the high prevalence of overweight and obese individuals in the sample (mean BMI = 29.97kg/m ^2^ ), which may have limited the power of the comparisons with those with a lower BMI.

The sleep parameters in the NHANES were not diagnosed sleep disorders, but rather self-reported parameters, related behaviors and complaints. The prevalences of sleep complaints (27.6%) and excessive daytime sleepiness (24.8%) were consistent with those reported in previous studies, 8 36 37 38 39 and they mirror the rates of conditions including insomnia and OSA. Despite the absence of objective sleep measures, these findings demonstrate that the critical role of sleep in overall human health has been underestimated.

Among the sleep-related variables, later chronotype (MSF _sc_ ) was linked to an elevated odd of cardiovascular, respiratory, and thyroid diseases. It is our belief that these results are not directly related to chronotype but, instead, reflect a consequence of the circadian misalignment commonly observed in individuals with later types. 15 40 They often experience misalignment between their timing system and occupational/societal obligations, including work and school schedules, leading to higher SJL, 15 41 which, together with sleep debt, has been shown to significantly increase sleepiness. 12 40 Disruptions in sleep patterns or circadian rhythms can cause endocrine alterations, as evidenced by the effect on thyroid hormones. 24 Moreover, the World Health Organization (WHO) has classified shift work as a probable carcinogen due to its effects on circadian rhythms, 42 despite being a topic in need of more exploration regarding its mechanisms and causality. Previous studies 25 26 using NHANES data have reported associations involving cancer and sleep disorders. Thus, it is reasonable to infer that individuals with later chronotypes may experience poorer sleep health, and that individuals whose sleep patterns are misaligned with societal norms face challenges in maintaining optimal health. 43

The evidence regarding the prevalence of sleep disorders and cancer is mixed. Approximately 60% (range: 15–99%) of cancer patients experience sleep disturbances, though not necessarily a diagnosis of a specific sleep disorder. 44 Having a sleep complaint has been associated with an increased likelihood of receiving a cancer diagnosis. 26 Sleep disorders seem to be involved to some extent as potential risk factors for specific cancers. Circadian rhythm disturbances have been linked to gastrointestinal, breast, prostate, thyroid, and lymphatic cancers, though the evidence for some is inconclusive. 42 45 46 47 The relationship between night shift work and cancer is particularly complex. Studies on breast and gastrointestinal tumors 42 48 report conflicting data and require further research to establish causal relationships. Insomnia is connected with breast, thyroid, liver, and lymphatic-system tumors 42 and it is reported three times more in cancer patients than in the general population. 49 Hypersomnia, particularly narcolepsy, has been related to head, neck, and gastric cancers, and subjectively-reported hypersomnia, to colorectal, liver, and lung cancers. 42 Similarly, OSA is correlated with a greater odd of developing melanoma and cancers of the breast, pancreas, and kidney, with preliminary evidence also showing it for prostate. 42 45 50 51 52 In the present study, the investigation did not center on specific cancer associations, primarily due to statistical criteria associated with sample size. This represents a significant gap in the extant literature that warrants further exploration.

The frequency of snoring, a potential indicator of OSA, was not related to any of the outcomes analyzed, except for cancer in the model adjusted for poverty ratio. Our analysis revealed an unexpected association between frequent snoring and a reduced probability of cancer, a finding that contrasts with those of mechanistic studies linking sleep fragmentation and OSA to tumorigenesis. 42 53 While lower socioeconomic status is generally connected with higher cancer incidence and poorer prognosis, 54 the results of the current study did not align with this pattern. We hypothesize that a masking effect may underlie these counterintuitive findings. Individuals with higher socioeconomic status could be more likely to have access to diagnostic services, which results in more frequent cancer detection. Conversely, individuals who regularly snore may engage more with the healthcare system, thereby creating a vicious cycle. This interpretation is corroborated by Ding et al. 55 (2024), who reported similar results for snoring and cancer using the same database. It is important to note that snoring does not necessarily indicate the presence of clinically-significant OSA, and it is possible that some cases are underreported, as this condition is often based on observations made by the individual's bed partner. The absence of physiological disturbances in snoring, such as hypoxia or sleep fragmentation, may not necessarily imply the same procarcinogenic likelihood related to moderate or severe OSA. Therefore, we advise caution against a causal interpretation and propose that these results may largely reflect complex healthcare-seeking behaviors and diagnostic biases within the cohort.

In contrast, a high frequency of breathing pauses or snoring events during sleep, which is a more strongly indicative sign of OSA, was associated with an increased odd of having cardiovascular and respiratory conditions. Meta-analyses have reported an elevated risk of general cardiovascular diseases, 56 57 hypertension, 58 and coronary heart disease 56 in OSA patients. Other studies 56 have demonstrated that individuals with moderate to severe OSA were more than twice as likely to experience stroke. Patients with chronic obstructive pulmonary disease often experience insomnia, OSA, and restless legs syndrome, which can severely affect their quality of life. 59 About 10% of OSA patients may also have heart block, with these episodes commonly tied to a reduction in blood oxygen levels by 4% or more. 60

Excessive daytime sleepiness (particularly when occurring with higher monthly frequency) and the reporting of sleep complaints resulted in an increased odds across all evaluated outcomes. This variable may be concurrent to multiple disruptions in healthy sleep patterns, including the presence of sleep disorders, circadian misalignment, and insufficient sleep duration, 61 62 which are all potential causes of non-restorative sleep. These findings are consistent with those of previous studies. It is well-established that low sleep efficiency is associated with a higher cardiovascular risk, 63 while insomnia has been linked to a higher incidence of myocardial infarction 64 and increased cardiovascular morbidity and mortality. 65 In addition, insufficient sleep duration and poor sleep quality were related to asthma risk. 66

Although the sleep parameters assessed in the present study were self-reported, this does not necessarily undermine the significance of the findings. Subjective aspects, including sleep quality and diurnal preferences, are crucial domains and complement objective measures of sleep. 67 The association between self-reported sleep complaints and negative health outcomes highlights the critical need for continued research in sleep health. These investigations could contribute to the prevention of serious diseases and help reduce morbidity and mortality rates. By showing that these interplays exist, even at the self-reported level, the inclusion of questions related to sleep in routine clinical assessments may be important in preventing chronic diseases.

The current cross-sectional analysis cannot establish causality or determine the directionality of the associations regarding impaired sleep health and chronic conditions. This is a crucial point, given the probable bidirectional relationship involving them. While the studied chronic diseases (or their treatments) can be a major source of sleep disturbances, 44 49 the reverse direction is also supported. 68 For instance, cancer has been demonstrated to alter the ghrelin-leptin pathway, 69 70 potentially altering orexinergic signalization and leading to hypersomnia. 42 Sleep fragmentation has been shown to intensify tumor growth in murine models. 53 On the other hand, Sillah et al. 50 demonstrated a 26% higher cancer incidence following a sleep apnea diagnosis, particularly for kidney, uterine, and breast cancers, as well as melanoma. This suggests that sleep alterations may also precede the development of cancer, potentially through mechanisms of chronic inflammation and impairment in antioxidant and immune defenses. 42 53

Some limitations to the present study should be acknowledged. Reliance on self-reported data from NHANES raises the potential for recall bias, a common limitation in such studies. The education level and comorbidities were not included in the analysis, although they are known to influence health disparities in the US. Despite these limitations, the current study makes a valuable contribution by leveraging a large, publicly-available dataset and applying a robust detailed methodology to the data. Thus, it provides a comprehensive analysis of multiple public health outcomes in the same population and underscores the significance of multiple sleep dimensions as key factors in health maintenance.

Our findings revealed a significant association involving excessive daytime sleepiness, sleep complaints, and a later chronotype, with an increased OR for all outcomes evaluated. Nearly 30% of the participants reported having trouble sleeping, while approximately 50% reported frequently snoring, 12%, stopping breathing or snorting, and 25% had excessive daytime sleepiness on at least half of the days of the month. These findings emphasize the potential health consequences of impaired sleep and highlight the importance of implementing strategies aimed at improving sleep health as part of the broader efforts to prevent and manage the conditions herein considered, particularly by addressing the problem of circadian misalignment.

One potential solution is the adoption of flexible work schedules that accommodate individuals' chronotype and sleep needs. Aligning working hours with individuals' circadian rhythms may mitigate the potential negative effects of circadian misalignment, such as fatigue, sleepiness, and reduced efficiency, and improve overall well-being and health outcomes. We recommend that healthcare professionals assess patients' complete sleep health dimensions, especially in those with a history of chronic diseases, and consider therapeutic interventions when sleep disorders are diagnosed.

## Conclusions

Increased odds of cardiovascular diseases, respiratory diseases, stroke, thyroid problems, and cancer were linked to reports of sleep complaints and excessive daytime sleepiness. An increased number of stopping breathing/snorting events, which may be a symptom of OSA, was related to cardiovascular and respiratory diseases, which indicates that OSA may be a key risk factor in health outcomes. Individuals with later chronotypes, indicated by the MSF _sc_ , had greater susceptibility to cardiovascular, respiratory, and thyroid diseases, emphasizing the potential effects of circadian factors on human physiology, and highlighting the significance of circadian misalignment regarding health.
