# Supplementary material for: Impact of Sleep Health Domains on Chronic Conditions: Findings from a Cross-Sectional Analysis Using Data from the NHANES 2017–2020
Source: Sleep Sci. 2026 Jul 17;19(2):1–10. doi: 10.1055/s-0046-1824545 (PMC13379272; doi:10.1055/s-0046-1824545)
Supplement: Supplementary file 1 — Supplementary Material [file 10-1055-s-0046-1824545-s250514-1.pdf]

	Models									
	CV disorders		RES disorders		Stroke		Thyroid problem		Cancer	
	VIF	*Toler*	*VIF*	*Toler*	*VIF*	*Toler*	*VIF*	*Toler*	*VIF*	*Toler*
Age	1.066	0.938	1.073	0.932	1.058	0.945	1.064	0.940	1.052	0.951
BMI	1.061	0.943	1.045	0.957	1.048	0.954	1.044	0.958	1.039	0.963
SJL	1.048	0.954	1.049	0.953	1.057	0.946	1.054	0.949	1.055	0.948
MSFsc	1.024	0.977	1.031	0.970	1.023	0.977	1.025	0.976	1.023	0.977
Gender	1.019	0.981	1.016	0.984	1.013	0.987	1.014	0.987	1.044	0.958
Frequency of snoring	1.083	0.923	1.095	0.913	1.075	0.930	1.081	0.925	1.074	0.931
Frequency of stop breathing/snorting	1.107	0.904	1.097	0.912	1.096	0.912	1.092	0.916	1.090	0.918
Reported a sleep problem	1.052	0.950	1.052	0.950	1.049	0.953	1.048	0.955	1.046	0.956
Excessive diurnal sleepiness	1.019	0.982	1.019	0.981	1.019	0.982	1.020	0.981	1.017	0.983
≥100 cigarrettes smoked during life	-	-	-	-	-	-	-	-	1.039	0.962

Abbreviations: BMI=body mass index; CI=confidence interval; CV=cardiovascular disorders; MSFsc=mid-sleep time on free days corrected for sleep debt on work days; RES=respiratory; SD=standard deviation; SJL=social jetlag; Toler=tolerance; VIF=variance inflation factor.

	Models adjusted for poverty income ratio									
	**CV disorders**		**RES disorders**		**Stroke**		**Thyroid problem**		**Cancer**	
	VIF	*Toler*	*VIF*	*Toler*	*VIF*	*Toler*	*VIF*	*Toler*	*VIF*	*Toler*
Age	1.073	0.932	1.079	0.927	1.071	0.934	1.070	0.934	1.056	0.947
BMI	1.061	0.942	1.045	0.957	1.051	0.952	1.044	0.958	1.037	0.964
SJL	1.048	0.955	1.050	0.953	1.054	0.949	1.054	0.949	1.057	0.946
MSFsc	1.024	0.976	1.031	0.970	1.025	0.976	1.025	0.976	1.023	0.977
Gender	1.020	0.980	1.017	0.983	1.015	0.985	1.014	0.986	1.044	0.958
Frequency of snoring	1.083	0.923	1.095	0.913	1.075	0.930	1.081	0.925	1.074	0.931
Frequency of stop breathing/snorting	1.106	0.904	1.097	0.912	1.095	0.913	1.093	0.915	1.090	0.917
Reported a sleep problem	1.053	0.950	1.053	0.950	1.054	0.949	1.048	0.954	1.044	0.958
Excessive diurnal sleepiness	1.019	0.981	1.020	0.981	1.020	0.980	1.020	0.981	1.017	0.983
Poverty income ratio	1.005	0.995	1.004	0.996	1.009	0.991	1.005	0.995	1.003	0.997
≥100 cigarrettes smoked during life	-	-	-	-	-	-	-	-	1.041	0.960

Abbreviations: BMI=body mass index; CI=confidence interval; CV=cardiovascular disorders; MSFsc=mid-sleep time on free days corrected for sleep debt on work days; RES=respiratory; SD=standard deviation; SJL=social jetlag; Toler=tolerance; VIF=variance inflation factor.
