# Supplementary material for: Impact of Sleep Health Domains on Chronic Conditions: Findings from a Cross-Sectional Analysis Using Data from the NHANES 2017–2020
Source: Sleep Sci. 2026 Jul 17;19(2):1–10. doi: 10.1055/s-0046-1824545 (PMC13379272; doi:10.1055/s-0046-1824545)
Supplement: Supplementary file 2 — Supplementary Material [file 10-1055-s-0046-1824545-s250514-2.pdf]

	Models			Models adjusted for poverty income ratio	
**Cardiovascular disorders**	**OR [95%CI]**	**p**	**Cardiovascular disorders**	**OR [95%CI]**	**p**
Age	1.08 [1.07; 1.09]	<0.001	*Age*	1.08 [1.07; 1.09]	<.001
BMI	1.04 [1.02; 1.05]	<0.001	*BMI*	1.04 [1.02; 1.05]	<.001
SJL	1.00 [0.97; 1.02]	0.734	*SJL*	1.00 [0.97; 1.02]	0.75
MSFsc	1.07 [1.02; 1.12]	0.007	*MSFsc*	1.07 [1.02; 1.12]	0.008
Gender			*Gender*		
Men – Women	2.18 [1.81; 2.62]	<0.001	Men – Women	2.19 [1.82; 2.64]	<.001
Frequency of snoring			*Frequency of snoring*		
≥3 – 0-2 (night(s)/week)	0.89 [0.74; 1.08]	0.257	≥3 – 0-2 (night(s)/week)	0.90 [0.74; 1.09]	0.271
Frequency of stop breathing/snorting			*Frequency of stop breathing/snorting*		
≥3 – 0-2 (night(s)/week)	1.31 [1.02; 1.68]	0.033	≥3 – 0-2 (night(s)/week)	1.31 [1.02; 1.68]	0.036
Told doctor had trouble sleeping			*Told doctor had trouble sleeping*		
Yes – No	1.75 [1.45; 2.12]	<0.001	Yes – No	1.75 [1.45; 2.11]	<.001
Excessive daytime sleepiness			*Excessive daytime sleepiness*		
16-30 – 0-4 (times/month)	1.50 [1.20; 1.88]	<0.001	16-30 – 0-4 (times/month)	1.95 [1.46; 2.59]	<.001
5-15 – 0-4 (times/month)	1.95 [1.47; 2.60]	<0.001	5-15 – 0-4 (times/month)	1.51 [1.20; 1.89]	<.001
			Poverty income ratio		
			Below poverty line – Equal or above porverty line	1.24 [0.96; 1.61]	0.102
**Respiratory disorders**			**Respiratory disorders**		
Age	1.00 [1.00; 1.01]	0.078	*Age*	1.00 [1.00; 1.01]	0.039
BMI	1.02 [1.01; 1.03]	<0.001	*BMI*	1.02 [1.01; 1.03]	<.001
SJL	0.98 [0.97; 0.99]	0.038	*SJL*	0.98 [0.97; 1.00]	0.049
MSFsc	1.08 [1.05; 1.12]	<0.001	*MSFsc*	1.08 [1.05; 1.12]	<.001

Gender		
Men – Women	0.85 [0.75; 0.95]	0.005
Frequency of snoring		
≥3 – 0-2 (night(s)/week)	1.02 [0.90; 1.16]	0.742
Frequency of stop breathing/snorting		
≥3 – 0-2 (night(s)/week)	1.46 [1.23; 1.73]	<0.001
Told doctor had trouble sleeping		
Yes – No	1.87 [1.65; 2.13]	<0.001
Excessive daytime sleepiness		
16-30 – 0-4 (times/month)	1.60 [1.39; 1.86]	<0.001
5-15 – 0-4 (times/month)	1.48 [1.21; 1.81]	<0.001
**Stroke**		
Age	1.07 [1.06; 1.07]	<0.001
BMI	1.01 [1.00; 1.03]	0.147
SJL	1.02 [0.99; 1.06]	0.252
MSFsc	1.02 [0.96; 1.09]	0.514
Gender		
Men – Women	1.19 [0.95; 1.50]	0.131
Frequency of snoring		
≥3 – 0-2 (night(s)/week)	0.98 [0.77; 1.25]	0.886
Frequency of stop breathing/snorting		

Gender		
Men – Women	0.85 [0.76; 0.96]	0.009
Frequency of snoring		
≥3 – 0-2 (night(s)/week)	1.02 [0.90; 1.16]	0.742
Frequency of stop breathing/snorting		
≥3 – 0-2 (night(s)/week)	1.46 [1.22; 1.73]	<.001
Told doctor had trouble sleeping		
Yes – No	1.87 [1.65; 2.12]	<.001
Excessive daytime sleepiness		
16-30 – 0-4 (times/month)	1.47 [1.20; 1.80]	<.001
5-15 – 0-4 (times/month)	1.61 [1.39; 1.86]	<.001
Poverty income ratio		
Below poverty line – Equal or above porverty line	1.28 [1.10; 1.50]	0.002
**Stroke**		
Age	1.07 [1.06; 1.08]	<.001
BMI	1.01 [1.00; 1.03]	0.14
SJL	1.02 [0.99; 1.06]	0.22
MSFsc	1.02 [0.96; 1.09]	0.552
Gender		
Men – Women	1.22 [0.97; 1.53]	0.093
Frequency of snoring		
≥3 – 0-2 (night(s)/week)	1.00 [0.78; 1.27]	0.977
Frequency of stop breathing/snorting		

≥3 – 0-2 (night(s)/week)	1.01 [0.73; 1.41]	0.931
Told doctor had trouble sleeping		
Yes – No	1.58 [1.25; 2.01]	<0.001
Excessive daytime sleepiness		
16-30 – 0-4 (times/month)	1.79 [1.36; 2.36]	<0.001
5-15 – 0-4 (times/month)	2.12 [1.50; 2.99]	<0.001
**Thyroid problem**		
Age	1.04 [1.03; 1.04]	<0.001
BMI	1.01 [1.00; 1.02]	0.072
SJL	0.97 [0.95; 0;99]	0.024
MSFsc	1.05 [1.01; 1.09]	0.027
Gender		
Men – Women	0.28 [0.24; 0.33]	<0.001
Frequency of snoring		
≥3 – 0-2 (night(s)/week)	0.94 [0.80; 1.11]	0.470
Frequency of stop breathing/snorting		
≥3 – 0-2 (night(s)/week)	1.00 [0.79; 1.28]	0.981
Told doctor had trouble sleeping		
Yes – No	1.43 [1.21; 1.68]	<0.001
Excessive daytime sleepiness		
16-30 – 0-4 (times/month)	1.23 [1.01; 1.50]	0.038
5-15 – 0-4 (times/month)	1.40 [1.08; 1.81]	0.012

≥3 – 0-2 (night(s)/week)	1.00 [0.72; 1.39]	0.985
Told doctor had trouble sleeping		
Yes – No	1.57 [1.23; 1.99]	<.001
Excessive daytime sleepiness		
16-30 – 0-4 (times/month)	2.09 [1.48; 2.96]	<.001
5-15 – 0-4 (times/month)	1.82 [1.38; 2.40]	<.001
Poverty income ratio		
Below poverty line – Equal or above porverty line	2.21 [1.66; 2.94]	<.001
**Thyroid problem**		
Age	1.04 [1.03; 1.04]	<.001
BMI	1.01 [1.00; 1.02]	0.069
SJL	0.97 [0.95; 1.00]	0.023
MSFsc	1.05 [1.01; 1.09]	0.028
Gender		
Men – Women	0.28 [0.24; 0.33]	<.001
Frequency of snoring		
≥3 – 0-2 (night(s)/week)	0.94 [0.80; 1.11]	0.459
Frequency of stop breathing/snorting		
≥3 – 0-2 (night(s)/week)	1.01 [0.79; 1.28]	0.961
Told doctor had trouble sleeping		
Yes – No	1.43 [1.21; 1.68]	<.001
Excessive daytime sleepiness		
16-30 – 0-4 (times/month)	1.40 [1.08; 1.81]	0.012
5-15 – 0-4 (times/month)	1.23 [1.01; 1.50]	0.039

**Cancer**		
Age	1.07 [1.06; 1.08]	<0.001
BMI	1.00 [0.98; 1.01]	0.620
SJL	1.00 [0.97; 1.02]	0.835
MSFsc	0.99 [0.94; 1.04]	0.558
Gender		
Men – Women	0.89 [0.75; 1.05]	0.171
Frequency of snoring		
≥3 – 0-2 (night(s)/week)	0.85 [0.71; 1.01]	0.062
Frequency of stop breathing/snorting		
≥3 – 0-2 (night(s)/week)	1.11 [0.87; 1.43]	0.399
Told doctor had trouble sleeping		
Yes – No	1.28 [1.07; 1.52]	0.007
Excessive daytime sleepiness		
16-30 – 0-4 (times/month)	1.29 [1.04; 1.59]	0.021
5-15 – 0-4 (times/month)	1.17 [0.87; 1.57]	0.299
Smoked unless 100 ciggarettes		
Yes – No	1.09 [0.92; 1.30]	0.293

Poverty income ratio		
Below poverty line – Equal or above porverty line	0.92 [0.74; 1.15]	0.452
**Cancer**		
Age	1.07 [1.06; 1.08]	<.001
BMI	1.00 [0.99; 1.01]	0.608
SJL	1.00 [0.97; 1.02]	0.839
MSFsc	0.98 [0.94; 1.03]	0.529
Gender		
Men – Women	0.88 [0.74; 1.04]	0.127
Frequency of snoring		
≥3 – 0-2 (night(s)/week)	0.84 [0.71; 1.;00]	0.049
Frequency of stop breathing/snorting		
≥3 – 0-2 (night(s)/week)	1.13 [0.88; 1.45]	0.357
Told doctor had trouble sleeping		
Yes – No	1.28 [1.07; 1.53]	0.007
Excessive daytime sleepiness		
16-30 – 0-4 (times/month)	1.18 [0.88; 1.58]	0.276
5-15 – 0-4 (times/month)	1.28 [1.03; 1.59]	0.025
Smoked unless 100 ciggarettes		
Yes – No	0.58 [0.44; 0.77]	<.001
Poverty income ratio		
Below poverty line – Equal or above porverty line		

Abbreviations: BMI=body mass index; CI=confidence interval; CV=cardiovascular disorders; MSFsc=mid-sleep time on free days corrected for sleep debt on work days; OR=odds ratio; SE=standard error; SJL=social jetlag.

Abbreviations: BMI=body mass index; CI=confidence interval; CV=cardiovascular disorders; MSFsc=mid-sleep time on free days corrected for sleep debt on work days; OR=odds ratio; SE=standard error; SJL=social jetlag.
